# #junkfluenced: the marketing of unhealthy food and beverages by social media influencers popular with Canadian children on YouTube, Instagram and TikTok

**DOI:** 10.1186/s12966-024-01589-4

**Published:** 2024-04-11

**Authors:** Monique Potvin Kent, Mariangela Bagnato, Ashley Amson, Lauren Remedios, Meghan Pritchard, Soulene Sabir, Grace Gillis, Elise Pauzé, Lana Vanderlee, Christine White, David Hammond

**Affiliations:** 1 School of Epidemiology and Public Health, University of Ottawa, Ottawa, ON Canada; 2 Interdisciplinary School of Health Sciences, University of Ottawa, Ottawa, ON Canada; 3 School of Nutrition, Centre NUTRISS (Nutrition, Santé, Société), Institute of Nutrition and Functional Foods, Université Laval, Québec, Québec Canada; 4 School of Public Health Sciences, University of Waterloo, Waterloo, ON Canada

**Keywords:** Unhealthy food marketing, Children, Social media influencers, Food environment, Digital media

## Abstract

**Background:**

Marketing of unhealthy foods to children on digital media significantly impacts their dietary preferences and contributes to diet-related noncommunicable diseases. Canadian children spend a significant amount of time on digital devices and are frequently exposed to unhealthy food marketing on social media, including by influencers with celebrity status who endorse products. This study aimed to examine the frequency, healthfulness, and power of unhealthy food marketing in posts by influencers popular with Canadian children on YouTube, Instagram and TikTok.

**Methods:**

The top 9 influencers popular amongst Canadian children aged 10–12 years were identified from the 2021 International Food Policy Study Youth Survey. A total of 2,232 Instagram, YouTube and TikTok posts made by these influencers between June 1st 2021 and May 31st 2022 were examined for instances of food marketing. Food products/brands were identified and frequencies were calculated for the number of posts promoting food products/brands, posts promoting products/brands classified as less healthy according to Health Canada’s Nutrient Profile Model (2018) and marketing techniques utilized.

**Results:**

YouTube had the highest average rate of food marketing instances per post, at a rate of 1 food marketing instance every 0.7 posts, while TikTok and Instagram had instances every 10.2 posts and 19.3 posts, respectively. Overall, fast food restaurants was the most promoted food category (21%), followed by regular soft drinks (13%), snacks (11%), candy and chocolate (11%) and water (8%). The most frequently used marketing techniques were appeals to fun/cool (37%), the use of songs or music (28%) and the product being consumed (25%). In terms of healthfulness, 83% of the products/brands (87% of brands and 82% of products) promoted were classified as less healthy.

**Conclusions:**

Social media influencers play a substantial role in promoting unhealthy food products to children, primarily fast food items. Given the significant impact of such marketing on children, there is a need for ongoing government-led monitoring, and it is crucial to include social media and influencer marketing in marketing restrictions targeting children in Canada to safeguard this vulnerable demographic.

**Supplementary Information:**

The online version contains supplementary material available at 10.1186/s12966-024-01589-4.

## Background

The marketing of food/beverages (herein referred to as “food”) high in fat, sugar and sodium directly affects children’s preferences for these foods and their consumption, increasing their risk of poor diet quality and obesity [1–6]. Children are vulnerable due to their stage of cognitive development, making it challenging for them to recognize the promotional intent of marketing [7–9]. Social media is a digital media platform increasingly being used by food marketers, which has proven to be impactful and cost-efficient for companies to market their products [10, 11].

According to recent Canadian evidence, total screen time for youth aged 10–17 years is 7.6 h per day on average, and other estimates have described that children aged 10–13 years spend over 3 h per day using digital devices during their leisure time [12, 13]. Children’s exposure to food marketing while using these devices is significant; one study estimated that children aged 7–11 years, on average, may be exposed to food marketing 1560 times annually on social media applications, and over 90% of featured products/brands are unhealthy [14]. An emerging tactic used by food companies to advertise their products on social media is the use of social media influencers. Social media influencers are online personas with large followings which may include musicians, actors, athletes, or individuals famous for producing social media content, who share their interests, recommendations and lives on social media with their followers by posting content including product and brand endorsements [15, 16]. They are often recognized by children as credible and trustworthy sources of information due to their celebrity-like status [15, 16]. Notably, children have reported that social media influencers are more “authentic” and “relatable” than other types of media content [17].

International evidence from the United Kingdom (UK), United States (US), Netherlands, Norway, and Spain has shown that social media influencers are a notable and powerful source of children’s exposure to unhealthy food marketing in digital media [17–21]. For instance, a study examining YouTube influencers popular with children aged 5–15 years in the UK found that 92.6% of their videos contained food cues with an estimated product placement rate of 29.9 cues/hour – a rate higher than that of television product placement [17]. Nearly half of these food cues were classified as “unhealthy” [17]. Similarly, data examining YouTube videos from the 5 most popular social media influencers with children in the US found that these videos were viewed about 1 billion times, with almost 50% of the videos containing food products and about 90% of products were classified as unhealthy [20].

Research has also demonstrated that social media influencers impact children’s behaviours. Two studies have demonstrated that children increased their intake of unhealthy foods after being exposed to unhealthy product promotions by social media influencers [15, 22]. Children also reported that vloggers (a blogger who shares their content in short videos) have an effect on their awareness and purchases of products and brands [18].

The World Health Organization (WHO) has recommended that unhealthy food marketing be restricted on social media and other digital spaces [23]. Currently in Canada, the industry self-regulatory initiative, the *Code for Children’s Food and Beverage Advertising, *designed to restrict unhealthy food advertising to children does not apply to social media [24]. The *Influencer Marketing Steering Committee for Ad Standards’* and the *Competition Act’s *guidelines on marketing disclosures for influencers also does not contain restrictions to protect children from unhealthy food marketing contained in influencer posts [25, 26]. Given the frequency of unhealthy food marketing cues in social media influencer posts and the impact of such marketing on children, it is essential to monitor this type of marketing to help inform policy. Very few studies have done this globally and most studies have focused on a single social media platform [17–22]. In Canada, no research has assessed the frequency of food marketing in social media influencer posts nor has it evaluated the healthfulness of the food products/brands featured in these posts. As a result, this study aimed to examine the frequency, healthfulness, and power (i.e. use of marketing techniques) of unhealthy food marketing in posts by social media influencers popular with Canadian children on YouTube, Instagram and TikTok.

## Methods

### Identification of social media influencers popular amongst children

Social media influencers that were most popular amongst children aged 10–12 years were extracted from the 2021 International Food Policy Study Youth Survey [27]. In this national sample of Canadian children, participants were asked “Who are your 3 favourite social media stars, TikTokers, or YouTubers?”. The top 5 most commonly reported influencers for both male and female respondents were identified. A total of 9 influencers were included, as one influencer was amongst the most popular influencers for both males and females (Table 1).

**Table 1:** Top 9 influencers amongst Canadian children 10–12 years

1	Addison Rae
2	Charli D’Amelio
3	DanTDM
4	Justin Bieber
5	Michou
6	Moriah Elizabeth
7	MrBeast
8	Squeezie
9	SSSniperWolf

### Collection of posts and content analysis

Posts (i.e. posted videos and photos but excluding stories) made by each of the 9 influencers between June 1st 2021 and May 31st 2022 on the 3 most popular social media applications amongst children were examined: Instagram, TikTok and YouTube [28]. Instagram is a platform for sharing mostly static pictures and short videos, whereas YouTube and TikTok are video-based platforms. All platforms are media sharing networks where you can follow or subscribe to other user’s posts. Social media posts were analyzed for instances of food marketing. Any instance where a branded food product is shown in the post was considered as ‘product marketing’, whereas ‘brand marketing’ referred to when a brand name/logo was shown or mentioned in the post, but the product was not shown. Due to the high frequency of posts on YouTube, a random sample (using a random choice generator) of 50% of YouTube videos were assessed and videos that were longer than 60 min were excluded. In addition, 50% of Instagram posts were assessed for influencers with over 300 posts (which applied to Justin Bieber only). Across all platforms, the 9 most popular influencers among children collectively shared 3,010 posts, of which 2,232 were examined (Table 2). Within this sample, 361 posts featured a food product or brand.

**Table 2:** Frequency of posts on YouTube, Instagram and TikTok between June 1st 2021 and May 31st 2022 examined amongst the nine most popular influencers for children 10–12 years old

Platform	Posts Collected n(%)	Posts Examined^a^ n(%)	Food Product & Brand Posts^a^ n(%)
YouTube	712(24)	354(16)	234(65)
Instagram	1306(43)	886(40)	39(11)
TikTok	992(33)	992(44)	88(24)
Total	**3010(100)**	**2232(100)**	**361(100)**

^a^A random sample of 50% of YouTube and Instagram posts were analyzed (posts examined)

Five research assistants identified instances of food marketing (i.e. any instance where a branded food product or brand is shown or mentioned in the post) within the social media posts and coded for the presence or absence of marketing techniques using a coding manual developed from previous work (See supplementary Table 1 for the list of marketing techniques and their defintions) [29]. The marketing techniques examined included the use of spokes characters (e.g. Tucan Sam) and celebrities (e.g. actors, athletes, etc.), and advertisement appeals such as appeals to fun/cool (e.g., depictions of the food in motion) and the use of appealing graphic effects (e.g., special effect explosions, cool fonts) among others. Inter-rater reliability was 94.5% and was calculated by comparing samples of posts coded by each research assistant before the coding began. Food products and brands were categorized into 18 food and beverage categories which were adapted from previous research by Potvin Kent et al. (Supplementary Table 2) [14, 30]. The food categories included: bread; sweet baked goods/desserts; candy and chocolate; breakfast cereal; dairy; meat and entrees (including fish, poultry, and meat products); fruit and vegetables; energy drinks; regular soft drinks; diet soft drinks; other sweetened beverages; water; snacks; fast food restaurants; non-fast food restaurants; food delivery services; condiments, spreads & dressings; and other (e.g. seasonings, non-dairy alternatives, etc.).

### Nutrient profiling

Health Canada’s proposed Nutrient Profile Model (NPM) from 2018 was used to categorize the healthfulness of each promoted product [31]. Nutrition information for each promoted product was collected in order of priority from the: 1) Canadian website of the food company; 2) website of a Canadian grocery store (e.g., Walmart Canada or Loblaws); 3) American website of the food company; 4) website of an American grocery store (e.g., Target) or; 5) international website of the food company or grocery store (e.g., L’Azur Gourmet). Healthfulness was determined using Health Canada’s defined thresholds for saturated fat, free sugars and sodium (Supplementary Table 3) to classify products that would be less healthy or “of concern from an advertising perspective”, versus healthier or “NOT of concern from an advertising perspective”. The nutritional data and food categorizations were reviewed by a Registered Dietitian (EP). Health Canada’s protocol for evaluating products with limited nutritional information was used to categorize some products (*n* = 54). Health Canada’s restaurant and food brands tool (unpublished) was used to categorize food brands and restaurants. Brands were considered less healthy if > 50% of the brand’s products were classified as “of concern from an advertising perspective” and considered healthier otherwise. Unlisted brands on the tool were excluded from the analysis (*n* = 22).

### Statistical analysis

Descriptive statistics were computed for Instagram, TikTok and YouTube. Frequencies were tabulated for the number of posts that promoted food products by product category, the number of posts that promoted food products/brands classified as less healthy, and the number of times marketing techniques were used in posts. Frequencies for YouTube, as well as Justin Bieber’s Instagram posts, were weighted to account for the 50% random sample (i.e. multiplied by 2). Rates of food marketing instances by platform were also calculated by dividing the total number of posts examined (Table 2) by the total number of food products/brands identified by platform (Table 3).

## Results

### Frequency of promoted food products and brands

A weighted total of 685 food products/brands (550 products and 135 brands) were promoted by the most popular influencers amongst children 10–12 years old between June 1st 2021 and May 31st 2022. Among social media platforms, YouTube had the highest rate of food marketing instances per post at a rate of 1 food marketing instance every 0.7 posts (meaning that, on average, there was more than one food marketing instance per post), compared to every 10.2 posts on TikTok and every 19.3 posts on Instagram (Table 3; by SMI in Supplementary Table 4).

**Table 3:** Weighted frequencies of food products/brands on YouTube, Instagram, and TikTok posts amongst the most popular influencers for children 10–12 years old between June 1st 2021 and May 31st 2022

Platform	Brand n(%)	Product n(%)	Total n(%)	Rate of food marketing/post
YouTube^a^	102(76)	440(80)	**542(79)**	**0.7**
Instagram^b^	20(15)	26(5)	**46(7)**	**19.3**
TikTok^c^	13(10)	84(15)	**97(14)**	**10.2**
Total	**135(100)**	**550(100)**	**685(100)**	**-**

^a^A random sample of 50% YouTube posts were analyzed and frequencies are weighted

^b^A random sample of 50% of Justin Bieber’s Instagram posts within the study time frame were analyzed and frequencies are weighted

^c^All TikTok posts collected within the study timeframe were analyzed

### Frequency of endorsed food products/brands by food category

Overall, among the food products/brands promoted by the most popular influencers amongst children 10–12 years old across YouTube, Instagram and TikTok, fast food restaurants was the most promoted food category (21%), followed by regular soft drinks (13%), snacks (11%), candy and chocolate (11%) and water (8%) (Table 4). On YouTube, fast food restaurants was the most promoted food category (21%), followed by regular soft drinks (16%), candy and chocolate (11%), snacks (11%) and condiments, spreads & dressings (9%). On Instagram, the most promoted food category was fast food restaurants (43%), followed by candy and chocolate (18%), other sweetened beverages (7%), water (11%) and other (11%). On TikTok, water was the most promoted food category (31%), followed by snacks (20%), and fast food restaurants (14%).

**Table 4:** Weighted frequencies of food categories on YouTube, Instagram, and TikTok posts amongst the most popular influencers for children 10–12 years old between June 1st 2021 and May 31st 2022

Food Category	YouTube^a^ n(%)	Instagram^b^ n(%)	TikTok^c^ n(%)	Total n(%)
Fast food restaurants	90(21)	12(43)	12(14)	**114(21)**
Regular soft drinks	70(16)	1(4)	1(1)	**72(13)**
Snacks	46(11)	0(0)	17(20)	**63(11)**
Candy and chocolate	48(11)	5(18)	7(8)	**60(11)**
Water	12(3)	3(11)	27(31)	**42(8)**
Condiments, spreads & dressings	38(9)	0(0)	2(2)	**40(7)**
Other	24(6)	3(11)	7(8)	**34(6)**
Sweet baked goods/desserts	24(6)	1(4)	7(8)	**32(6)**
Other sweetened beverages	18(4)	2(7)	4(5)	**24(4)**
Meat and entrees (including fish, poultry, and meat products)	16(4)	0(0)	1(1)	**17(3)**
Breakfast cereal	14(3)	0(0)	0(0)	**14(3)**
Dairy	10(2)	0(0)	1(1)	**11(2)**
Energy drinks	8(2)	1(4)	1(1)	**10(2)**
Diet soft drinks	8(2)	0(0)	0(0)	**8(1)**
Bread	6(1)	0(0)	0(0)	**6(1)**
Fruits and vegetables	2(0)	0(0)	0(0)	**2(0)**
Total	**434(100)**	**28(100)**	**87(100)**	**549(100)**

^a^A random sample of 50% YouTube posts were analyzed and frequencies are weighted

^b^A random sample of 50% of Justin Bieber’s Instagram posts within the study time frame were analyzed and frequencies are weighted

^c^All TikTok posts collected within the study timeframe were analyzed

In terms of the top branded food products featured in posts by the most popular influencers amongst children, MyMuse water (9%), Coca-Cola (5%) and Dunkin Donut iced coffee (4%) were featured the most overall. By platform, on YouTube the most featured products were Coca-Cola (6%) and McDonald’s fries (5%), while on Instagram, TimBiebs were featured the most (31%), and on TikTok, MyMuse enhanced water (32%) and Dunkin Donut iced coffee (12%) were the most featured products. In terms of the top food brands featured in posts overall, amongst both product and brand advertising by the most popular influencers amongst children, McDonalds (9%), Coca-Cola (7%) and MyMuse (5%) were featured the most overall (Supplementary Table 5). By platform, on YouTube the most featured brands were McDonalds (11%), Coca-Cola (8%) and Starbucks (5%), while on Instagram, Tim Hortons (22%) and Starbucks (7%) were featured the most, and on TikTok, MyMuse (31%), Takis (19%) and Dunkin Donuts (12%) were the most featured brands.

### Frequency of marketing techniques featured in posts

Amongst social media posts containing food products/brands shared by the most popular influencers for children aged 10–12 years overall, the most frequently used marketing technique was appeals to fun/cool (37% of posts), followed by the use of songs/music (28%) and the product being consumed (25%) (Table 5). Several techniques were not observed, and techniques such as the presence of children or teens, use of licensed or spokes characters and calls-to-action were rarely used. On YouTube, the most frequently used technique was appeals to fun/cool (44%), followed by product consumed (23%) and the use of songs/music (20%). On Instagram, the most used techniques were viral marketing (38%), appeals to fun/cool (31%) and the use of other influencers (26%). On TikTok, the most used technique was the use of songs or music (64%), followed by product consumed (36%) and viral marketing (27%).

**Table 5:** Weighted frequencies of marketing techniques used in posts containing food products/brands on YouTube, Instagram, and TikTok amongst the most popular influencers for children 10–12 years old between June 1st 2021 and May 31st 2022

Marketing Technique	YouTube^a^ *N* = 234 n(%)*	Instagram^b^ *N* = 39 n(%)*	TikTok^c^ *N* = 88 n(%)*	Total *N* = 361 n(%)*
Appeals to fun/cool	102(44)	12(31)	21(24)	**135(37)**
Songs or music	46(20)	0(0)	56(64)	**102(28)**
Product consumed	54(23)	5(13)	32(36)	**91(25)**
Unusual product appearance	34(15)	2(5)	12(14)	**48(13)**
Viral marketing	6(3)	15(38)	24(27)	**45(12)**
Use of other influencers	16(7)	10(26)	14(16)	**40(11)**
Appealing graphic effects	12(5)	9(23)	14(16)	**35(10)**
Sponsorship disclosure	6(3)	5(13)	17(19)	**28(8)**
Presence of teens	14(6)	3(8)	4(5)	**21(6)**
Unusual product flavour	8(3)	2(5)	10(11)	**20(6)**
Appeals to beauty	0(0)	2(5)	14(16)	**16(4)**
Appeals to healthfulness	4(2)	4(10)	5(6)	**13(4)**
Calls-to-action	2(1)	6(15)	5(6)	**13(4)**
Presence of children	10(4)	1(3)	0(0)	**11(3)**
Incentives/giveaways	2(1)	1(3)	4(4)	**7(2)**
Spokes characters	6(3)	1(3)	0(0)	**7(2)**
Appeals to achievement	2(1)	1(3)	3(3)	**6(2)**
Use of actors	6(3)	0(0)	0(0)	**6(2)**
Adult-child situations	4(2)	0(0)	1(1)	**5(1)**
Appeals to athleticism	2(1)	2(5)	0(0)	**4(1)**
Animations	2(1)	0(0)	1(1)	**3(0)**
Licensed characters	2(1)	0(0)	1(1)	**3(0)**
Mention of child	2(1)	1(3)	0(0)	**3(0)**
Use of athletes	2(1)	1(3)	0(0)	**3(0)**
Appeals to energy	0(0)	1(3)	1(1)	**2(0)**
Appeals to sex	0(0)	1(3)	1(1)	**2(0)**
Child or teen language	2(1)	0(0)	0(0)	**2(0)**
Child themes	0(0)	1(3)	1(1)	**2(0)**
Teen themes	2(1)	0(0)	0(0)	**2(0)**
Limited time item/seasonal	0(0)	0(0)	1(1)	**1(0)**
Other cartoon characters	0(0)	0(0)	1(1)	**1(0)**
Price promotions	0(0)	0(0)	1(1)	**1(0)**
Advercation	0(0)	0(0)	0(0)	**0(0)**
Adult-teen situations	0(0)	0(0)	0(0)	**0(0)**
Appeals to social enhancement	0(0)	0(0)	0(0)	**0(0)**
Corporate social responsibility	0(0)	0(0)	0(0)	**0(0)**
Cross-promotions	0(0)	0(0)	0(0)	**0(0)**
Games or activities	0(0)	0(0)	0(0)	**0(0)**
Gender of child specified	0(0)	0(0)	0(0)	**0(0)**
Gender of teen specified	0(0)	0(0)	0(0)	**0(0)**
Mention of teen	0(0)	0(0)	0(0)	**0(0)**
Use of musicians	0(0)	0(0)	0(0)	**0(0)**

^a^A random sample of 50% YouTube posts were analyzed and frequencies are weighted

^b^A random sample of 50% of Justin Bieber’s Instagram posts within the study time frame were analyzed and frequencies are weighted

^c^All TikTok posts collected within the study timeframe were analyzed

^*^% are a proportion of total posts containing food products/brands (N)

### Healthfulness of endorsed food products/brands

Overall, across the examined social media platforms between June 1st 2021 and May 31st 2022, 83% of the products/brands (82% of products and 87% of brands) promoted by the most popular social media influencers amongst children aged 10–12 years were classified as less healthy (Table 6). On YouTube, Instagram and TikTok, 84%, 85% and 53% of products/brands were classified as less healthy, respectively.

**Table 6:** Healthfulness of food products/brands on YouTube, Instagram, and TikTok posts amongst the most popular influencers for children 10–12 years old between June 1st 2021 and May 31st 2022

	**Brands** **n(%)**	**Products** **n(%)**	**Total** **n(%)**
YouTube			
Healthier	14(16)	52(12)	**66(13)**
Less healthy	72(84)	390(88)	**462(88)**
Total	**86(100)**	**442(100)**	**528(100)**
Instagram			
Healthier	0(0)	6(23)	**6(15)**
Less healthy	15(100)	20(77)	**35(85)**
Total	**15(100)**	**26(100)**	**41(100)**
TikTok			
Healthier	0(0)	43(50)	**43(47)**
Less healthy	5(100)	43(50)	**48(53)**
Total	**5(100)**	**86(100)**	**91(100)**
Total			
Healthier	14(13)	101(18)	**115(17)**
Less healthy	92(87)	453(82)	**545(83)**
Total	**106(100)**	**554(100)**	**660(100)**

^a^A random sample of 50% YouTube posts were analyzed and frequencies are weighted

^b^A random sample of 50% of Justin Bieber’s Instagram posts within the study time frame were analyzed and frequencies are weighted

^c^All TikTok posts collected within the study timeframe were analyzed

## Discussion

This research provides insight into the nature and healthfulness of food products/brands promoted by SMIs popular with Canadian children and the marketing techniques used across 3 popular social media platforms. Food products/brands high in fat, sugar and sodium and their unhealthy product categories, like fast food and sugar-sweetened beverages, are frequently endorsed in posts by social media influencers popular amongst Canadian children, and over one third of all promoted products/brands contained appeals to fun/cool (e.g., depictions of the food in motion).

### Frequency and healthfulness of food marketing

In a span of one year, between June 2021 and May 2022, the top 9 influencers popular amongst 10–12 year old Canadian children promoted 685 food products/brands. YouTube had the highest number of food marketing instances identified, totalling 542 products/brands (79% of total) promoted over the 1-year period at a rate of 1 food marketing instance every 0.7 posts. These results are consistent with comparable literature from the US which found that the estimated frequency of food cues within influencer posts was also high, at an estimated rate of 260 cues over 8 months (13 influencers examined) [32]. Even higher rates of food cues were found in the UK where 1,045 branded food product and food retail cues over 1 year (2 influencers examined) were reported [17]. Such high levels of food influencer marketing on YouTube is worrisome, considering the notable power these influencers have over children’s consumption and brand preferences, as well as YouTube’s traction amongst this population [17, 18]. YouTube is used extensively by children, and food marketing rates may be higher on this platform compared to others due to its popularity amongst youth [18, 28]. It is also worth noting that although we examined social media influencers popular with children in Canada, many of these same influencers are popular in other countries including in the United States therefore the potential impact and reach of marketing by these influencers is significant.

Fast food restaurants (21%) and sugar-sweetened beverages (regular soft-drinks and other sweetened beverages; 17%) were the most frequently endorsed food categories across social media platforms overall. The prominence of these categories, in addition to other frequently endorsed categories like candy and chocolate and snacks, has also been noted in similar studies [17, 32, 33]. We also found that 83% of endorsed products/brands were classified as less healthy, according to Health Canada’s proposed NPM, which is comparable to similar studies and broader research examining both social media and traditional media [17, 32]. The ubiquitous marketing of foods that fall under these unhealthy categories poses a well-established risk to the short- and long-term health of children [11, 15, 34, 35]. Youth are being #junkfluenced by SMIs.

### The power of marketing instances

Across all 3 social media platforms, the post making appeals to fun/cool was the most common technique. Other common techniques were the use of songs/music, the product being consumed, and viral marketing (i.e. prompting sharing or interacting with the post), all used to target young audiences and boost engagement with the post or account [36]. The prominence of product consumption and exhibiting food products/brands alongside influencers is concerning, as it likely cues positive awareness and attitudes towards these products amongst children [37–42]. Product/brand endorsements by these social media influencers is also a marketing technique in itself, as children often consider these influencers to be celebrities and highly credible [16, 32].

Although children have difficulty recognizing the difference between entertainment and marketing and may benefit from a disclosure to help them distinguish the two, sponsorship disclosures were only used in about 8% of total marketing instances [7–9]. This is consistent with other studies investigating advertising viewed by both children and adolescents, which also found that any present sponsorship disclosures were discreet [17, 43–45]. However, such messages may help them recognize this content as marketing and may decrease the child’s desirability towards the product/brand [46, 47]. Although disclosures may be helpful to improve recognition, they would be unlikely to act as a reasonable alternative to restrictions since their effectiveness depends on their design/prominence and the industry may find ways to attenuate their effect [48]. It is worth noting that both Canadian-based and US-based influencers are legally required by the Competition Bureau’s *Competition Act *and the Federal Trade Commission, respectively, to disclose any material connections they have with brands they are promoting [26, 49]. Although we cannot be certain if all product endorsements are a result of “material connections”, it is possible that many social media influencers are not complying with federal regulations [26, 49].

### Strengths & limitations

To our knowledge, this study is the first internationally to examine food marketing among social media influencers popular with children across three platforms: YouTube, Instagram and TikTok. Although this study only focused on the SMIs popular amongst Canadian children, these results are potentially applicable internationally, as these SMIs are likely to be popular amongst children in other countries as well. In terms of limitations, the estimated food marketing rates computed encompass children’s potential exposure to food marketing among social media influencers, and not their actual measured exposure. We also only exclusively examined posts and did not include product endorsements on Instagram stories, which may have led us toad underestimate the frequency of food marketing instances. Additionally, the comparison of the calculated rate of posts per food marketing instance by social media platform should be interpreted with caution, as posts on various platforms vary considerably in length. For instance, video posts on YouTube may be 30 min in length, while Instagram posts consists mostly of photos. Future research should collect the length of posts in seconds (for video posts only) to compute these rates as a function of time and allow for more accurate comparisons between platforms. We are also not aware of whether these influencers were paid to promote these products or not. For feasibility reasons, the content analysis was conducted with only a 50% random sample of posts for YouTube due to lengthy videos (that exceeded 60 min), and Instagram posts made by influencers with more than 300 posts (i.e. Justin Bieber). Frequency weights were used to account for this, so the resulting number of marketing instances are estimates. Additionally, the nutritional information for some products, mostly those for fast food restaurants, were not available and were consequently classified using assumptions proposed by Health Canada’s NPM for products with limited nutrition information. Since Health Canada’s nutrient criteria are stringent and most items sold by fast food restaurants would be considered “of concern” as per the NPM, it is unlikely that items with missing nutrition information were misclassified. In terms of brands, about one third were considered missing, as they could not be classified using Health Canada’s NPM. Lastly, a social media influencer promoting a product is likely to be perceived as “cool” by children, but this was not accounted for in the classification of posts as appealing to fun/cool. As a result, the frequency of this appeal across social media platforms (37%) may be an underestimation. Future research should investigate the impact these SMI promotions are having on children’s purchase intentions and consumption.

### Policy implications

Children are frequent users of social media, and digital food marketing is more powerful, engaging and often viewed as entertainment compared to marketing in traditional media platforms such as television [12, 50]. Social media influencers are an established tactic used by marketers to increase the appeal and children’s positive attitudes towards their products/brands due to their celebrity status [16, 46]. This status, in addition to persuasive marketing techniques, increases the authentic appeal of the promotions, making it difficult for children to notice they are being marketed to [16, 46].

To protect children from unhealthy food marketing by SMIs on social media, government led-mandatory restrictions are needed. YouTube, the most popular social media platform amongst children, which had the highest rate of food marketing per post in our study, banned food ads in and around content ‘made for children’ in 2020 [18, 32]. However, such restrictions have shown to be ineffective as influencer marketing is excluded [32]. Official platform age restrictions are also ineffective at deterring children from accessing social media platforms and related social media influencer content [51]. Recent research from the UK has shown that even though children must be over the age of 13 to create a social media profile on most platforms, 69% of 8–12 year old’s report being between the ages of 13–15 on their user profile [52]. Government-led legislation or regulations that restrict unhealthy food marketing by social media influencers are key to protect children’s health. Such regulations need to include a broad range of social media influencers beyond those typically thought of as appealing to children, as children follow a broad range of influencers, as demonstrated in our study. Monitoring by government of all aspects of social media marketing, including food marketing by social media influencers who are and are not primarily directed at or appealing to children, is also vital to understanding the dynamic nature of unhealthy marketing in this platform and better inform policies that protect children in Canada and globally.

## Conclusions

Popular social media influencers were a significant source of children’s exposure to unhealthy food marketing on YouTube, Instagram and TikTok. Overall, the majority of products/brands promoted by social media influencers popular among children were considered to be less healthy according to Health Canada, and fast food restaurants dominated promotions. In addition to the posts being shared by influential social media influencers, the promoted products/brands often contained appeals to fun/cool. Due to the high levels of exposure to this unhealthy marketing and the impact social media influencers have on children, continued monitoring of this type of marketing is warranted. It is also paramount that social media and influencer marketing are included in government-led marketing restrictions to children in Canada and globally to protect this vulnerable population.

### Supplementary Information

**Additional file 1: Supplementary Table 1.** Coding manual for marketing techniques. **Supplementary Table 2. **Food product/brand categories based on previous research. **Supplementary Table 3. **Nutrient thresholds for Health Canada’s proposed Nutrient Profile Model. **Supplementary Table 4.** Weighted frequencies of food products/brands in YouTube, Instagram, and TikTok posts amongst the most popular influencers for children 10-12 years old between June 1^st^ 2021 and May 31^st^ 2022. **Supplementary Table 5.** Weighted frequencies of the top 3 food products/brands in YouTube, Instagram, and TikTok posts amongst the most popular influencers for children 10-12 years old between June 1^st^ 2021 and May 31^st^ 2022.

## Data Availability

The datasets used and analyzed for this study can be made available from the corresponding authors on reasonable request.

## Abbreviations

UK: United Kingdom
US: United States
NPM: Nutrient Profile Model

## References

[1] Glanz K, Sallis JF, Saelens BE, Frank LD (2005). Healthy Nutrition Environments: Concepts and measures. Am J Health Promot.

[2] Swinburn BA, Sacks G, Hall KD, McPherson K, Finegood DT, Moodie ML (2011). The global obesity pandemic: Shaped by global drivers and local environments. Lancet.

[3] McGinnis JM, Gootman JA, Kraak VI (2006). Food marketing to children and youth: Threat or opportunity? Washington.

[4] Cairns G, Angus K, Hastings G, Caraher M (2013). Systematic reviews of the evidence on the nature, extent and effects of food marketing to children A retrospective summary. Appetite.

[5] Sadeghirad B, Duhaney T, Motaghipisheh S, Campbell NR, Johnston BC (2016). Influence of unhealthy food and beverage marketing on children’s dietary intake and preference: A systematic review and meta-analysis of randomized trials. Obes Rev.

[6] Norman J, Kelly B, Boyland E, McMahon A-T (2016). The impact of marketing and advertising on food behaviours: Evaluating the evidence for a causal relationship. Curr Nutr Rep.

[7] Wilcox BL (2004). Report of the APA task force on advertising and children. PsycEXTRA Dataset.

[8] John DR (1999). Consumer socialization of children: A retrospective look at twenty-five years of research. J Consumer Res.

[9] Carter OBJ, Patterson LJ, Donovan RJ, Ewing MT, Roberts CM (2011). Children’s understanding of the selling versus persuasive intent of junk food advertising: Implications for regulation. Soc Sci Med.

[10] Pechmann C, Levine L, Loughlin S, Leslie F (2005). Impulsive and self-conscious: Adolescents’ vulnerability to advertising and promotion. J Public Policy Mark.

[11] Mc Carthy CM, de Vries R, Mackenbach JD. The influence of unhealthy food and beverage marketing through social media and advergaming on diet‐related outcomes in children—a systematic review. Obes Rev. 2022;23(6). 10.1111/obr.13441. 10.1111/obr.13441 PMC9286387 35301815

[12] Brisson-Boivin, K. The Digital Well-Being of Canadian Families. MediaSmarts. Ottawa. 2018. https://mediasmarts.ca/sites/mediasmarts/files/publication-report/full/digital-canadian-families.pdf. Accessed 22 Sept 2023.

[13] Demers-Potvin É, White M, Potvin Kent M, Nieto C, White CM, Zheng X (2022). Adolescents’ media usage and self-reported exposure to advertising across six countries: Implications for less healthy food and beverage marketing. BMJ Open.

[14] Potvin Kent M, Pauzé E, Roy E, de Billy N, Czoli C. Children and adolescents’ exposure to food and beverage marketing in social media apps. Pediatr Obes. 2019;14(6). 10.1111/ijpo.12508. 10.1111/ijpo.12508 PMC6590224 30690924

[15] Smit CR, Buijs L, van Woudenberg TJ, Bevelander KE, Buijzen M. The impact of social media influencers on children’s dietary behaviors. Front Psychol. 2020;10. 10.3389/fpsyg.2019.02975. 10.3389/fpsyg.2019.02975 PMC6967733 31998202

[16] De Veirman M, Hudders L, Nelson MR. What is influencer marketing and how does it target children? A review and direction for future research. Front Psychol. 2019;10. 10.3389/fpsyg.2019.02685. 10.3389/fpsyg.2019.02685 PMC6901676 31849783

[17] Coates AE, Hardman CA, Halford JC, Christiansen P, Boyland EJ. Food and beverage cues featured in YouTube videos of Social Media Influencers popular with children: An exploratory study. Front Psychol. 2019;10. 10.3389/fpsyg.2019.02142. 10.3389/fpsyg.2019.02142 PMC6763597 31616344

[18] Folkvord F, Bevelander KE, Rozendaal E, Hermans R. Children’s bonding with popular YouTube vloggers and their attitudes toward brand and product endorsements in vlogs: An explorative study. Young Consumers. 2019;20(2). 10.1108/yc-12-2018-0896.

[19] Young and Exposed to Unhealthy Marketing: Digital food marketing using influencers. Forbrukerradet. 2019. https://fil.forbrukerradet.no/wp-content/uploads/2019/02/young-and-exposed-to-unhealthy-marketing-digital-food-marketing-using-influencers-report-february-2019.pdf. Accessed 26 Sept 2023.

[20] Alruwaily A, Mangold C, Greene T, Arshonsky J, Cassidy O, Pomeranz JL, et al. Child social media influencers and unhealthy food product placement. Pediatrics. 2020;146(5). 10.1542/peds.2019-4057 10.1542/peds.2019-4057 PMC7786816 33106342

[21] Martínez-Pastor E, Vizcaíno-Laorga R, Atauri-Mezquida D. Health-related food advertising on Kid YouTuber vlogger channels. Heliyon. 2021;7(10). 10.1016/j.heliyon.2021.e08178. 10.1016/j.heliyon.2021.e08178 PMC8551517 34746464

[22] Coates AE, Hardman CA, Halford JC, Christiansen P, Boyland EJ. The effect of influencer marketing of food and a “protective” advertising disclosure on children’s Food Intake. Pediatr Obes. 2019;14(10). 10.1111/ijpo.12540. 10.1111/ijpo.12540 31168959

[23] World Health Organization – Europe. Monitoring And Restricting Digital Marketing Of Unhealthy Products To Children And Adolescents. World Health Organization. 2018. https://www.who.int/europe/publications/i/item/WHO-EURO-2019-3592-43351-60815. Accessed 7 Sept 2023.

[24] The Association of Canadian Advertisers. Code for the Responsible Advertising of Food and Beverage Products to Children (“Food and Beverage Advertising Code”). Available from: https://acaweb.ca/en/wp-content/uploads/sites/2/2023/05/FoodAndBeverageAdvertisingCode-FINAL-20230505-1.pdf. Cited 2023 Sept 28.

[25] Ad Standards. Influencer Marketing Steering Committee: Disclosure Guidelines. 2020.. Available from: https://adstandards.ca/wp-content/uploads/Ad-Standards-Influencer-Marketing-Steering-Committee-Disclosure-Guidelines_FALL2020_EN.pdf. Cited 2023 Sept 28.

[26] Competition Bureau Canada. Government of Canada. Government of Canada; 2022. Available from: https://ised-isde.canada.ca/site/competition-bureau-canada/en/deceptive-marketing-practices/types-deceptive-marketing-practices/influencer-marketing-and-competition-act. Cited 2023 Dec 4.

[27] International Food Policy Study – 2021 Youth Survey. Methods. Available from: https://foodpolicystudy.com/methods/. Cited 2023 Sept 11.

[28] Vogels EA. Teens, social media and technology 2022. Pew Research Center; 2022. Available from: https://www.pewresearch.org/internet/2022/08/10/teens-social-media-and-technology-2022/#:~:text=YouTube%20tops%20the%202022%20teen,six%2Din%2Dten%20teens. Cited 2023 Dec 4.

[29] Amson A, Pauzé E, Remedios L, Pritchard M, Potvin KM (2022). Adolescent exposure to food and beverage marketing on social media by gender: A pilot study. Public Health Nutr.

[30] Potvin Kent M, Pauzé E (2018). The effectiveness of self-regulation in limiting the advertising of unhealthy foods and beverages on children’s preferred websites in Canada. Public Health Nutr.

[31] Health Canada. Monitoring food marketing to children: A protocol for classifying foods. Version 1.0. 2021. Unpublished.

[32] Fleming‐Milici F, Phaneuf L, Harris J. Prevalence of food and beverage brands in “made‐for‐kids” child‐influencer YouTube videos: 2019–2020. Pediatr Obes. 2023;18(4). 10.1111/ijpo.13008. 10.1111/ijpo.13008 36755375

[33] Amson A, Pauzé E, Remedios L, Pritchard M, Potvin KM (2022). Adolescent exposure to food and beverage marketing on social media by gender: A pilot study. Public Health Nutr.

[34] Packer J, Russell SJ, Siovolgyi G, McLaren K, Stansfield C, Viner RM (2022). The impact on dietary outcomes of celebrities and influencers in marketing unhealthy foods to children: A systematic review and meta-analysis. Nutrients.

[35] Smith R, Kelly B, Yeatman H, Boyland E (2019). Food marketing influences children’s attitudes, preferences and consumption: A systematic critical review. Nutrients.

[36] Truman E, Elliott C. Identifying food marketing to teenagers: A scoping review. Int J Behav Nutr Phys Act. 2019;16(1). 10.1186/s12966-019-0833-2. 10.1186/s12966-019-0833-2 PMC6700978 31426809

[37] Cairns G (2019). A critical review of evidence on the sociocultural impacts of Food Marketing and policy implications. Appetite.

[38] Naderer B, Matthes J, Zeller P (2017). Placing snacks in children’s movies: Cognitive, evaluative, and CONATIVE effects of product placements with character product interaction. Int J Advert.

[39] Coates AE, Hardman CA, Halford JC, Christiansen P, Boyland EJ (2020). “it’s just addictive people that make addictive videos”: Children’s understanding of and attitudes towards influencer marketing of food and beverages by YouTube video bloggers. Int J Environ Res Public Health.

[40] Martínez C, Olsson T (2018). Making sense of youtubers: How Swedish children construct and negotiate the YouTuber Misslisibell as a girl celebrity. J Child Media.

[41] Martínez C, Jarlbro G, Sandberg H (2013). Children’s views and practices regarding online advertising. Nordicom Rev.

[42] Thaichon P, Quach TN (2016). Online marketing communications and childhood’s intention to consume unhealthy food. Australas Mark J.

[43] Winzer E, Naderer B, Klein S, Lercher L, Wakolbinger M (2022). Promotion of food and beverages by German-speaking influencers popular with adolescents on TikTok, YouTube and Instagram. Int J Environ Res Public Health.

[44] Qutteina Y, Hallez L, Mennes N, De Backer C, Smits T. What do adolescents see on social media? A diary study of Food Marketing Images on social media. Front Psychol. 2019;10. 10.3389/fpsyg.2019.02637 10.3389/fpsyg.2019.02637 PMC6883917 31824391

[45] van der Bend DL, Jakstas T, van Kleef E, Shrewsbury VA, Bucher T. Adolescents’ exposure to and evaluation of food promotions on social media: A multi-method approach. Int J Behav Nutr Phys Act. 2022;19(1). 10.1186/s12966-022-01310-3. 10.1186/s12966-022-01310-3 PMC9235222 35761362

[46] Boerman SC, van Reijmersdal EA. Disclosing influencer marketing on YouTube to children: The moderating role of para-social relationship. Front Psychol. 2020;10. 10.3389/fpsyg.2019.03042. 10.3389/fpsyg.2019.03042 PMC6985591 32038405

[47] van der Bend DLM, Gijsman N, Bucher T, Shrewsbury VA, van Trijp H, van Kleef E (2023). Can I @handle it? the effects of sponsorship disclosure in TikTok influencer marketing videos with different product integration levels on adolescents’ persuasion knowledge and brand outcomes. Comput Hum Behav.

[48] Brüns JD, Meißner M (2023). Show me that you are advertising: Visual salience of products attenuates detrimental effects of persuasion knowledge activation in influencer advertising. Comput Hum Behav.

[49] Office of the Federal Register. Part 255.5 - Disclosure of material connections. 2023. Available from: https://www.ecfr.gov/current/title-16/chapter-I/subchapter-B/part-255/section-255.5. Cited 2023 Dec 4.

[50] Montgomery KC, Chester J, Grier SA, Dorfman L (2012). The new threat of Digital Marketing. Pediatr Clin North Am.

[51] Sacks G, Looi ES (2020). The advertising policies of major social media platforms overlook the imperative to restrict the exposure of children and adolescents to the promotion of unhealthy foods and beverages. Int J Environ Res Public Health.

[52] Ofcom Research & Intelligence. Children’s Online User Ages 2023: Quantitative Research Study. Ofcom. 2023. https://www.ofcom.org.uk/__data/assets/pdf_file/0022/272281/childrens-user-age-2023-chart-pack.pdf. Accessed 20 Mar 2024.

