# Supplementary material for: #junkfluenced: the marketing of unhealthy food and beverages by social media influencers popular with Canadian children on YouTube, Instagram and TikTok
Source: Int J Behav Nutr Phys Act. 2024 Apr 11;21:37. doi: 10.1186/s12966-024-01589-4 (PMC11010392; doi:10.1186/s12966-024-01589-4)
Supplement: Supplementary file 1 — Additional file 1: Supplementary Table 1. Coding manual for marketing techniques. Supplementary Table 2. Food product/brand categories based on previous research. Supplementary Table 3. Nutrient thresholds for Health Canada’s proposed Nutrient Profile Model. Supplementary Table 4. Weighted frequencies of food products/brands in YouTube, Instagram, and TikTok posts amongst the most popular influencers for children 10-12 years old between June 1st 2021 and May 31st 2022. Supplementary Table 5. Weighted frequencies of the top 3 food products/brands in YouTube, Instagram, and TikTok posts amongst the most popular influencers for children 10-12 years old between June 1st 2021 and May 31st 2022. [file 12966_2024_1589_MOESM1_ESM.docx]

**Supplementary Table 1.** Coding manual for marketing techniques

| **Name of Technique** | **Description and examples** | **Coding Variable** |
| --- | --- | --- |
| Post ID |  | Same ID number as in ad dataset so that the two datasets can be linked |
| Brand or product |  | 1 – Brand  2 - Product |
| Name of brand |  | Open text |
| Name of product |  | Open text |
| Product type | Defines what type of product the food item is. Different flavours of a product are all considered one product (e.g., Birthday cake Oreos and Mint chocolate Oreos both considered as just Oreos).  Any fast food item is automatically coded as fast food restaurants (e.g., McDouble burger is coded as fast food restaurants). | 1 – Bread  2 – Sweet baked goods/desserts  3 – Candy & chocolate  4 – Breakfast cereal  5 – Dairy  6 – Meat & entrees (including fish, poultry, meat products)  7 – Fruits and vegetables  8 – Energy drinks  9 – Soft drinks (regular)  10 – Soft drinks (diet)  11 – Other sweetened beverages (e.g., sweetened coffee/tea, hot chocolate, fruit drinks, etc.)  12 – Water  13 – Snacks  14 – Fast food restaurant  15 – Non-fast food restaurants (i.e., sit down)  16 – Food delivery services  17 – Condiments, spreads & dressings  18 – Other |
| Unusual product appearance | Ad depicts a product’s shape or colour that is unconventional or unusual for that specific product. E.g., If goldfish crackers (typically fish shaped and yellow in color) come out with a rainbow-coloured version of their product | 1 – no  2 – yes |
| Unusual product flavour | Ad promotes a product flavour that is unconventional or unusual for that specific product, or a flavour that is not a ‘real’ or ‘discernable’ flavour. E.g., Coca-Cola coming out with cherry flavoured Coke. This could also include the presentation of the flavour in a ‘negative’ way that may appeal to youth - tastes crazy, weird, sour, wacky | 1 – no  2 – yes |
| Product consumed | Does the influencer actually consume the product/brand? (e.g., drink or eat) | 1 – no  2 – yes |
| **Sponsorship disclosure** | | |
| Sponsorship disclosure | The influencer displays or mentions a sponsorship disclosure. E.g., “This product is brought to you by McDonald’s/X”, or “I’ve teamed up with Tim Hortons to bring you...”. If not said verbally, usually there is a hashtag tied to the sponsorship or something may be written in the description. This needs to be explicit. Even if the influencer is obviously in a partnership with the brand (E.g., Justin Bieber and Tim Hortons), if they do not say that the ad is sponsored, then it is not considered a sponsorship disclosure. | 1 – no  2 – yes |
| **Reference to others** | | |
| Presence of children | Child is present in the ad  A child is anyone under the age of 12. | 1 – no  2 – yes |
| Mention of child | The influencer refers to a child who is not physically present with them – whether it is a statement “this product is great for kids”  A child is anyone under the age of 12. | 1 – no  2 – yes |
| Gender of child specified | Ad dominantly displays the product/brand alongside a specific gender of a child (e.g., girls jumping rope while drinking chocolate milk) | 1 – no  2 – yes |
| Presence of teen | Teen is present in the ad | 1 – no  2 – yes |
| Mention of teen | The influencer refers to a teenager who is not physically present with them – whether it is a statement “this product is great for teens”  A teenager is anyone between 13-17 years old. | 1 – no  2 – yes |
| Gender of teen specified | Ad dominantly displays the product /brand alongside a specific gender of a teen (e.g., boys skateboarding while drinking an energy drink) | 1 – no  2 – yes |
| Adult-child situation | Post features situations that play on the parent-child relationship or other authority-based relationship (e.g., coach-child, teacher-child). | 1 – no  2 – yes |
| Adult-teen situation | Post features situations that play on the parent-teen relationship or other authority-based relationship (e.g., coach-teen, teacher-teen). | 1 – no  2 – yes |
| Child or teen language | Post uses language that is associated with children, that is frequently used by children, or that is directed at children.  E.g., “OMG”, “dope” etc. | 1 – no  2 – yes |
| **Themes** | | |
| Child themes | Post uses themes, designs, colours, images, or other elements of audiovisual design that are commonly associated with children, such as fantasy, magic, mystery, suspense, adventure, zoo animals, virtual worlds, etc. This could include references to, or the incorporation of, popular trends in children’s interests of preferences, which may vary year-to-year or based on geographic location. **This needs to be a deliberate inclusion of child themes to market the product.** | 1 – no  2 – yes |
| Teenager themes | Post uses themes, designs, colours, images, or other elements of audiovisual design that are commonly associated with teens, such as themes linked to high school, social media, ‘hanging-out’, popularity, fashion, risk-taking, independence, etc. This could include references to, or the incorporation of, popular trends in teens interests or preferences, which may vary year-to-year or based on geographic location. **This needs to be a deliberate inclusion of teen themes to market the product.** | 1 – no  2 – yes |
| **Characters** | | |
| Spokes characters | A fictional/cartoon character that is defined by a set of human attributes and characteristics to give the brand a unique personality. E.g., Tony the Tiger, Pillsbury Doughboy, etc. | 1 – no  2 – yes |
| Licensed characters | A licensed character involves licensing the rights from the owner of the cartoon character to place images on a product. E.g., using Spiderman on a Luncheables package. | 1 – no  2 – yes |
| Other cartoon characters that are not spokes or licensed | Post features generic cartoon characters, cartoon children/teens, animals, or imaginary/virtual creatures etc. that are not branded or licensed characters, celebrities, or cross-promotions to other media. | 1 – no  2 – yes |
| Use of Athletes | An athlete(s) is present or referenced in the post. E.g., Lebron James, Hailey Wickenheiser, etc. | 1 – no  2 – yes |
| Use of Actors | An actor(s) is present or referenced in the post. E.g., Chris Hemsworth, Natalie Portman, etc. | 1 – no  2 – yes |
| Use of Musicians | A musician(s) is present or referenced in the post. E.g., Ed Sheeran, Ariana Grande, etc. | 1 – no  2 – yes |
| Use of other Influencers | Another influencer is present or referenced in the post. E.g., Charli D’Amelio featuring Addison Rae, MrBeast featuring Jake Paul, etc. | 1 – no  2 – yes |
| **Appeals** | | |
| Appeals to fun or cool | Post makes appeals to the food or beverage item being fun or funny, having fun while eating the product, being happy, humour or coolness/novelty. This could include depictions of the food itself doing something fun, or depictions of the food in motion, for example, a cookie diving into milk, or candies ‘exploding’ out of ice cream, juggling food products, someone spinning Oreos on their finger, etc. It can also be part of the name such as “Fun Dip” or “Kool Kreatures”. | 1 – no  2 – yes |
| Appeals to social enhancement | The post highlights the product’s ability to enhance making friends, peer acceptance, or being social with others. E.g., Coca-Cola advertisements that feature people giving friends Coke’s with their names on them. | 1 – no  2 – yes |
| Appeals to athleticism | The post highlights the product’s attributes to athleticism, referring to its ability to boost one’s strength, speed, or sports performance or features individual(s) doing athletic activities with the product.  E.g., People snowboarding while drinking Mountain Dew. | 1 – no  2 – yes |
| Appeals to sex | The post includes aspects of romance, sex, or sexuality to market a product. E.g., Paris Hilton wearing a bikini while eating a Carl’s Jr Burger. | 1 – no  2 – yes |
| Appeals to beauty | The post includes aspects of beauty or attractiveness to market the product. E.g., An influencer doing their make-up while consuming a drink or food product. | 1 – no  2 – yes |
| Appeals to healthfulness | Post makes explicit appeals related to the healthfulness or nutritional quality of the product, its ability to promote wellness, growth, strength, or physical activity. The post can also make implicit appeals to health or nutrition, such as the product being displayed alongside “healthy foods” (e.g., fresh fruit being depicted in an ad for breakfast cereal), or the product being shown consumed by children while participating in physical activity. E.g., the post includes health and nutrition claims/symbols, as well as claims or symbols referring to the product being organic or natural. | 1 – no  2 – yes |
| Appeals to energy | The influencer references or utilizes energy as a selling point e.g., “Red bull gives you wings” | 1 – no  2 – yes |
| Appeals to achievement or success | The post highlights the product’s ability to help with achievement or accomplishment or the influencer indicates this product has helped them become successful. E.g., Mentos that show problem solving or achievement because of consuming the product; Grey Poupon showing status or achievement. | 1 – no  2 – yes |
| **Effects** | | |
| Appealing graphic effects | Visual effects are used in the post or to market the product. E.g., explosions, lights, fast cutting, slow motion, dynamic images, etc. Graphic imagery is used in the post or to enhance the display of the product. E.g., bright colours, eye-catching backgrounds, fonts, etc.  **i.e., eye-catching elements** | 1 – no  2 – yes |
| Songs or music | Music is used in the post. E.g., songs, jingles, sound effects, etc. | 1 – no  2 – yes |
| Animations | Animations are used in the post to make the product appealing. E.g., the use of cartoons to interact with the product. | 1 – no  2 – yes |
| **Promotions** | | |
| Cross promotions | Post features cross-promotions to movies/sporting events/TV shows etc. other than one of the types of characters or celebrities described above. E.g., the ad features aspects of a well-known fictional world, without specifically including the fictional characters.  Note: these may appear in addition to the presence of any characters described above.  **Does NOT include promotion of other food or non-food brands (e.g., Uber Eats) or to other food products.** | 1 – no  2 – yes |
| Price promotions | Post includes a price-promotion or premium, including discounted prices on other merchandise included with the purchase of a food or beverage product. E.g., Charlie D’Amelio offers a discount on makeup if you buy Takis. | 1 – no  2 – yes |
| Incentives and giveaways | Post promotes contests, prizes, or giveaways available with or without purchase. E.g., Addison Rae will give away $1000 to the first 100 people that comment on her post. | 1 – no  2 – yes |
| Calls to action | Post encourages consumers to either participate in a campaign, visit a product/brand/company website, social media, or games-based brand website or promote opportunities to “join”, “become a member”, complete a quiz, poll, or survey. Encourage the user to spend more time with the brand. E.g., Charli D’Amelio will encourage you to check out her Linktree and sign up for an account.  **I.e., sending the user/viewer to an additional website or link** | 1 – no  2 – yes |
| Corporate responsibility | Post makes appeals to sustainability, philanthropy or contributing to other social causes. E.g., For every like on this post I (the influencer) will donate a dollar to cleaning up beaches. | 1 – no  2 – yes |
| Viral marketing | Prompts viewers to engage with the brand by commenting, replying, sharing information with their peers (peer-to-peer marketing), re-posting content to their own feeds, tagging friends, or using specific hashtags. E.g., “Be sure to share this post with all of your friends”.  A hashtag of a company is displayed either in the description, comments section, or on the screen.  E.g., #wendys #invisalign.  Includes tagging brands or other influencers (>10k followers)  Emphasis is on **sharing with others** | 1 – no  2 – yes |
| Games | Presence of games or activities within the post (including on packaging, marketing display, etc.). E.g., MrBeast is playing a video game while drinking Coke. Also includes tie-ins with games (e.g., UNO deal with Pizza Pizza) | 1 – no  2 – yes |
| Advercation | Ad is linked to online educational content. E.g., a chocolate ad where the influencer teaches you about where the cocoa beans came from and how they were harvested, etc. | 1 – no  2 – yes |
| Limited time item/seasonal | Is product promoted as a limited time or seasonal item? (e.g., “Starbucks Peppermint Mocha only here for the holiday season!”) | 1 – no  2 – yes |

**Supplementary Table 2.** Food product/brand categories based on previous research

|  | **Food Category** | **Definition** |
| --- | --- | --- |
| 1 | Bread | Bread from bakeries (manufacturers who primarily make baked goods), bread loaves and rolls, bread dough, crescent rolls |
| 2 | Sweet baked goods/desserts | Cakes, cookies, sweet biscuits, muffins, donuts, pies, tarts, snack cakes, sweet rolls, pudding, flavoured gelatin, icing, ice cream, frozen yogurt & treats, pastries, waffles, pancakes, French toast |
| 3 | Candy & chocolate | Candy without chocolate, chocolate bars, candy with chocolate, boxed chocolates, confectionary manufacturers (e.g., lollipops, cotton candy, etc.) |
| 4 | Breakfast cereal | Cold or hot cereals (e.g., Frosted Flakes, Quaker Instant Oatmeal) |
| 5 | Dairy | Cheese, dairy product images, milk, milk powder, milkshakes, yogurt, cheese spreads |
| 6 | Meat & entrees (including fish, poultry, meat products) | Entrees, fish & canned shellfish, luncheon meat, wieners & franks (e.g., hotdogs), pasta meals, pizza & pizza rolls |
| 7 | Fruits & vegetables | Fruit: canned, dried, fresh, frozen, vegetables: canned fresh, frozen, packaged |
| 8 | Energy Drinks | Energy drinks and, energy drink brands (e.g., Red Bull, Monster, Guru) |
| 9 | Soft drinks (regular) | Carbonated non-diet soft drinks that do not contain alcohol (e.g., Coke, Pepsi) |
| 10 | Soft drinks (diet) | Diet soft drinks that do not contain alcohol (e.g., Coke Zero, Diet Pepsi) |
| 11 | Other sweetened beverages | Juices, drinks & nectars, powder & liquid milk flavourings, sports drinks, sweetened coffee/tea, hot chocolate, flavoured waters (e.g., Vitamin Water) |
| 12 | Water | Bottled water (excluding water softeners & additives), sparkling or natural, enhanced waters (e.g., My Muse Enhanced Water) |
| 13 | Snacks | Portable snacks (e.g., granola bars, fruit-based snacks, toaster pastries, dried fruit), snack crackers, soda crackers, snack foods (e.g., chips, popcorn, pretzels, nuts, jerky), compartment snacks and lunch kits (e.g., Lunchables) |
| 14 | Fast food restaurant | Restaurants where food is ordered and purchased at a counter with limited to no table service (e.g., McDonalds, Subway) and their products |
| 15 | Non-fast food restaurant | Sit down restaurants with table service (e.g., Milestones, Red Lobster) and their products |
| 16 | Food delivery services | Food or grocery delivery services (e.g., Uber Eats, Skip the Dishes, Good Food, Hello Fresh, Instacart) |
| 17 | Condiments, spreads & dressings | Condiments (e.g., ketchup, mayonnaise, maple syrup), oils (e.g., olive oil, cooking sprays, margarine), spreads (e.g., peanut butter, Nutella, jams) (excluding cheese spreads) & salad dressings |
| 18 | Other | Seasonings, & toppings, non-dairy alternatives (e.g., non-dairy milk, plant-based cheese), natural food supplements (e.g., protein powder) food manufacturers and exporters (e.g., George Weston Limited, Saputo Inc.) |

**Supplementary Table 3.** Nutrient thresholds for Health Canada’s proposed Nutrient Profile Model

|  | **Nutrient** | **Thresholds for foods** | **Thresholds for main dishes with a RA above 200g** |
| --- | --- | --- | --- |
| **Low in** | Saturated Fat | A total of 2 g SFA per RA or serving of stated size, whichever is the greater and ≤ 15% energy from the SFA | A total of 2 g SFA per 100g and ≤ 15% energy is from the SFA |
|  | Sodium | 140 mg per RA or serving of stated size whichever is the greater or 140 mg per 50 g of the product if the RA is ≤ 30g or 30 mL | 140 mg per 100g |
|  | Sugars | 5 g per RA or serving of stated size whichever is the greater or 5 g per 50 g of the product if the RA is ≤ 30g or 30 mL | 5 g per 100g |
| *Source: Health Canada. Health Canada's Proposed Nutrient Profile Model for Restricting Marketing to Children. 2019. Unpublished [cited 2023 July 19]. | | | |

**Supplementary Table 4.** Weighted frequencies of food products/brands in YouTube, Instagram, and TikTok posts amongst the most popular influencers for children 10-12 years old between June 1^st^ 2021 and May 31^st^ 2022

| **YouTube** | | | |
| --- | --- | --- | --- |
| **Influencer** | **Brands**  **n(%)** | **Products**  **n(%)** | **Brands/Products**  **n(%)** |
| **Addison Rae** | 0(0) | 0(0) | **0(0)** |
| **Charli D’Amelio** | 0(0) | 14(3) | **14(3)** |
| **DanTDM** | 2(2) | 0(0) | **2(0)** |
| **Justin Bieber** | 0(0) | 0(0) | **0(0)** |
| **Michou** | 12(12) | 44(10) | **56(10)** |
| **Moriah Elizabeth** | 0(0) | 0(0) | **0(0)** |
| **MrBeast** | 0(0) | 8(2) | **8(1)** |
| **Squeezie** | 0(0) | 8(2) | **8(1)** |
| **SSSniperWolf** | 88(86) | 366(83) | **454(84)** |
| **Total** | **102(100)** | **440(100)** | **542(100)** |
| **Instagram** | | | |
| **Influencer** | **Brands**  **n(%)** | **Products**  **n(%)** | **Brands/Products**  **n(%)** |
| **Addison Rae** | 5(25) | 5(19) | **10(22)** |
| **Charli D’Amelio** | 6(30) | 7(27) | **13(28)** |
| **DanTDM** | 0(0) | 0(0) | **0(0)** |
| **Justin Bieber** | 6(30) | 10(38) | **16(35)** |
| **Michou** | 1(5) | 0(0) | **1(2)** |
| **Moriah Elizabeth** | 0(0) | 0(0) | **0(0)** |
| **MrBeast** | 1(5) | 4(15) | **5(11)** |
| **Squeezie** | 0(0) | 0(0) | **0(0)** |
| **SSSniperWolf** | 1(5) | 0(0) | **1(2)** |
| **Total** | **20(100)** | **26(100)** | **46(100)** |
| **TikTok** | | | |
| **Influencer** | **Brands**  **n(%)** | **Products**  **n(%)** | **Brands/Products**  **n(%)** |
| **Addison Rae** | 1(8) | 3(4) | **4(4)** |
| **Charli D’Amelio** | 6(46) | 71(85) | **77(79)** |
| **DanTDM** | 0(0) | 0(0) | **0(0)** |
| **Justin Bieber** | 0(0) | 0(0) | **0(0)** |
| **Michou** | 3(23) | 1(1) | **4(4)** |
| **Moriah Elizabeth** | 0(0) | 3(4) | **3(3)** |
| **MrBeast** | 3(23) | 6(7) | **9(9)** |
| **Squeezie** | 0(0) | 0(0) | **0(0)** |
| **SSSniperWolf** | 0(0) | 0(0) | **0(0)** |
| **Total** | **13(100)** | **84(100)** | **97(100)** |
| **Total** | | | |
| **Influencer** | **Brands**  **n(%)** | **Products**  **n(%)** | **Brands/Products**  **n(%)** |
| **Addison Rae** | 6(4) | 8(1) | **14(2)** |
| **Charli D’Amelio** | 12(9) | 92(17) | **104(15)** |
| **DanTDM** | 2(1) | 0(0) | **2(0)** |
| **Justin Bieber** | 6(4) | 10(2) | **16(2)** |
| **Michou** | 16(12) | 45(8) | **61(9)** |
| **Moriah Elizabeth** | 0(0) | 3(1) | **3(0)** |
| **MrBeast** | 4(3) | 18(3) | **22(3)** |
| **Squeezie** | 0(0) | 8(1) | **8(1)** |
| **SSSniperWolf** | 89(66) | 366(67) | **455(66)** |
| **Total** | **135(100)** | **550(100)** | **685(98)** |

^a^ A random sample of 50% YouTube posts were analyzed and frequencies are weighted.

^b^ A random sample of 50% of Justin Bieber’s Instagram posts within the study time frame were analyzed and frequencies are weighted.

^c^ All TikTok posts collected within the study timeframe were analyzed.

**Supplementary Table 5.** Weighted frequencies of the top 3 food products/brands in YouTube, Instagram, and TikTok posts amongst the most popular influencers for children 10-12 years old between June 1^st^ 2021 and May 31^st^ 2022

| **YouTube** | | | | |
| --- | --- | --- | --- | --- |
| **Ranking** | **Brand** | **n(%)** | **Product** | **n(%)** |
| **1** | McDonald’s | 60(11) | Coca-Cola | 28(6) |
| **2** | Coca-Cola | 46(8) | McDonald’s fries | 20(5) |
| **3** | Starbucks | 28(5) | Coca-Cola zero | 10(2) |
| **3** |  |  | Pepsi | 10(2) |
| **Instagram** | | | | |
| **Ranking** | **Brand** | **n(%)** | **Product** | **n(%)** |
| **1** | Tim Hortons | 10(22) | TimBiebs | 8(31) |
| **2** | Starbucks | 3(7) | San Pellegrino sparkling water | 2(8) |
|  |  |  | Dunkin Donut iced coffee | 2(8) |
|  |  |  | Feastables chocolate bars | 2(8) |
| **TikTok** | | | | |
| **Ranking** | **Brand** | **n(%)** | **Product** | **n(%)** |
| **1** | MyMuse | 30(31) | MyMuse enhanced water | 27(32) |
| **2** | Takis | 18(19) | Dunkin Donut iced coffee | 10(12) |
| **3** | Dunkin Donuts | 12(12) | Takis rolled tortilla chips | 6(7) |
| **Total** | | | | |
| **Ranking** | **Brand** | **n(%)** | **Product** | **n(%)** |
| **1** | McDonald’s | 65(9) | MyMuse enhanced water | 29(9) |
| **2** | Coca-Cola | 47(7) | Coca-Cola | 15(5) |
| **3** | MyMuse | 33(5) | Dunkin Donut iced coffee | 13(4) |

^a^ A random sample of 50% YouTube posts were analyzed and frequencies are weighted.

^b^ A random sample of 50% of Justin Bieber’s Instagram posts within the study time frame were analyzed and frequencies are weighted.

^c^ All TikTok posts collected within the study timeframe were analyzed.
